# Crowdsourced assessment of common genetic contribution to predicting anti-TNF treatment response in rheumatoid arthritis

**DOI:** 10.1038/ncomms12460

**Published:** 2016-08-23

**Authors:** Solveig K. Sieberts, Fan Zhu, Javier García-García, Eli Stahl, Abhishek Pratap, Gaurav Pandey, Dimitrios Pappas, Daniel Aguilar, Bernat Anton, Jaume Bonet, Ridvan Eksi, Oriol Fornés, Emre Guney, Hongdong Li, Manuel Alejandro Marín, Bharat Panwar, Joan Planas-Iglesias, Daniel Poglayen, Jing Cui, Andre O. Falcao, Christine Suver, Bruce Hoff, Venkat S. K. Balagurusamy, Donna Dillenberger, Elias Chaibub Neto, Thea Norman, Tero Aittokallio, Muhammad Ammad-ud-din, Chloe-Agathe Azencott, Víctor Bellón, Valentina Boeva, Kerstin Bunte, Himanshu Chheda, Lu Cheng, Jukka Corander, Michel Dumontier, Anna Goldenberg, Peddinti Gopalacharyulu, Mohsen Hajiloo, Daniel Hidru, Alok Jaiswal, Samuel Kaski, Beyrem Khalfaoui, Suleiman Ali Khan, Eric R. Kramer, Pekka Marttinen, Aziz M. Mezlini, Bhuvan Molparia, Matti Pirinen, Janna Saarela, Matthias Samwald, Véronique Stoven, Hao Tang, Jing Tang, Ali Torkamani, Jean-Phillipe Vert, Bo Wang, Tao Wang, Krister Wennerberg, Nathan E. Wineinger, Guanghua Xiao, Yang Xie, Rae Yeung, Xiaowei Zhan, Cheng Zhao, Members of the Rheumatoid Arthritis Challenge Consortium, Manuel Calaza, Haitham Elmarakeby, Lenwood S. Heath, Quan Long, Jonathan D. Moore, Stephen Obol Opiyo, Richard S. Savage, Jun Zhu, Jeff Greenberg, Joel Kremer, Kaleb Michaud, Anne Barton, Marieke Coenen, Xavier Mariette, Corinne Miceli, Nancy Shadick, Michael Weinblatt, Niek de Vries, Paul P. Tak, Danielle Gerlag, Tom W. J. Huizinga, Fina Kurreeman, Cornelia F. Allaart, S. Louis Bridges Jr., Lindsey Criswell, Larry Moreland, Lars Klareskog, Saedis Saevarsdottir, Leonid Padyukov, Peter K. Gregersen, Stephen Friend, Robert Plenge, Gustavo Stolovitzky, Baldo Oliva, Yuanfang Guan, Lara M. Mangravite

**Affiliations:** 1 Sage Bionetworks, Seattle, Washington 98115, USA; 2 Department of Computational Medicine and Bioinformatics, University of Michigan, Ann Arbor, Michigan 48109, USA; 3 Structural Bioinformatics Group (GRIB/IMIM), Departament de Ciències Experimentals i de la Salut, Universitat Pompeu Fabra, Barcelona 08003, Spain; 4 Center for Statistical Genetics, Division of Psychiatric Genomics, Icahn School of Medicine at Mount Sinai, New York, New York 10029, USA; 5 Icahn Institute for Genomics and Multiscale Biology, Icahn School of Medicine at Mount Sinai, New York, New York 10029, USA; 6 Department of Genetics and Genomic Sciences, Icahn School of Medicine at Mount Sinai, New York, New York 10029, USA; 7 Division of Rheumatology, Department of Medicine, Columbia University, New York, New York 10032, USA; 8 Corrona LLC, Southborough, Massachusetts 01772, USA; 9 Center for Complex Network Research, Northeastern University, Boston, Massachusetts 02115, USA; 10 Center for Cancer Systems Biology, Dana-Farber Cancer Institute, Boston, Massachusetts 02215, USA; 11 Division of Rheumatology, Immunology, and Allergy, Brigham and Women's Hospital, Harvard Medical School, Boston, Massachusetts 02115, USA; 12 Department of Informatics, Faculty of Sciences, University of Lisbon, Lisbon 1749-016, Portugal; 13 IBM T.J. Watson Research Center, Yorktown Heights, New York, New York 10598, USA; 14 Institute for Molecular Medicine Finland (FIMM), University of Helsinki, Helsinki FI-00014, Finland; 15 Department of Computer Science, Aalto University, Espoo 02150, Finland; 16 Helsinki Institute for Information Technology (HIIT), Esbo 02150, Finland; 17 MINES ParisTech, PSL-Research University, CBIO-Centre for Computational Biology, Fontainebleau 77300, France; 18 Institut Curie, Paris 75248, France; 19 Bioinformatics, Biostatistics, Epidemiology and Computational Systems Biology of Cancer, INSERM U900, Paris 75248, France; 20 Department of Mathematics and Statistics, University of Helsinki, Helsinki FI-00014, Finland; 21 Stanford Center for Biomedical Informatics, Stanford University, Stanford, California 94305, USA; 22 Department of Computer Science, University of Toronto, Toronto, Ontario, Canada M5G OA5; 23 Genetics and Genome Biology, SickKids Research Institute, Toronto, Ontario, Canada M5G 0A4; 24 Department of Computer Science, University of Helsinki, Helsinki FI-00014, Finland; 25 Department of Integrative Structural and Computational Biology, The Scripps Translational Science Institute, The Scripps Research Institute, La Jolla, California 92037, USA; 26 Center for Medical Statistics, Informatics, and Intelligent Systems, Medical University of Vienna, Vienna 1090, Austria; 27 Quantitative Biomedical Research Center, University of Texas Southwestern Medical Center, Dallas, Texas 75390, USA; 28 Department of Computer Science, Stanford University, Stanford, California 94305, USA; 29 Harold C. Simmons Comprehensive Cancer Center, University of Texas Southwestern Medical Center, Dallas, Texas 75390, USA; 30 Department of Paediatrics, Department of Immunology, Institute of Medical Sciences, University of Toronto, Toronto, Ontario, Canada M5S 1A8; 31 Cell Biology, SickKids Research Institute, Toronto, Ontario, Canada M5G 0A4; 32 Center for the Genetics of Host Defense, University of Texas Southwestern Medical Center, Dallas, Texas 75390, USA; 33 Department of Medicine, New York University School of Medicine, New York, New York 10003, USA; 34 Department of Medicine, Division of Rheumatology, Albany Medical College, Albany, New York 12206, USA; 35 Department of Medicine, University of Nebraska Medical Center, Omaha, Nebraska 68198, USA; 36 National Data Bank for Rheumatic Diseases, Wichita, Kansas 67214, USA; 37 Arthritis Research UK Centre for Genetics and Genomics, Centre for Musculoskeletal Research, Manchester Academic Health Sciences Centre, The University of Manchester, Manchester M13 9PT, UK; 38 NIHR Manchester Musculoskeletal Biomedical Research Unit, Central Manchester Foundation Trust, Manchester M13 9WU, UK; 39 Department of Human Genetics, Radboud University Nijmegen Medical Centre, Nijmegen 6525 GA, The Netherlands; 40 Department of Rheumatology, Université Paris-Sud, Orsay 91400, France; 41 APHP–Hôpital Bicêtre, Center of Immunology of Viral Infections and Autoimmune Diseases (IMVA) INSERM U1184, Paris 94276, France; 42 Department of Clinical Immunology and Rheumatology, Academic Medical Center/University of Amsterdam, Amsterdam 1105 AZ, The Netherlands; 43 Department of Medicine, Cambridge University, Cambridge CB2 1TN, UK; 44 Department of Rheumatology, Ghent University, Ghent 9000, Belgium; 45 GlaxoSmithKline, Stevenage SG1 2NY, UK; 46 Clinical Unit, GlaxoSmithKline, Cambridge CB2 0QQ, UK; 47 Department of Rheumatology, Leiden University Medical Centre, Leiden 2300 RC, The Netherlands; 48 Division of Clinical Immunology and Rheumatology, Department of Medicine, University of Alabama at Birmingham, Birmingham, Alabama 35294, USA; 49 Rosalind Russell/Ephraim P Engleman Rheumatology Research Center, Division of Rheumatology, Department of Medicine, University of California San Francisco, San Francisco, California 94143, USA; 50 Division of Rheumatology and Clinical Immunology, University of Pittsburgh, Pittsburgh, Pennsylvania 15261, USA; 51 Rheumatology Unit, Department of Medicine, Karolinska Hospital and Karolinska Institutet, Solna 171 76 Stockholm, Sweden; 52 Robert S. Boas Center for Genomics and Human Genetics, Feinstein Institute for Medical Research, North Shore LIJ Health System, Manhasset, New York 11030, USA.; 53 Merck Research Labs, Merck and Co., Inc., Boston, Massachusetts 02115, USA; 54 Division of Clinical Immunology and Rheumatology, Department of Medicine, University of Alabama at Birmingham, Birmingham, Alabama 35294, USA; 55 Department of Medicine, Division of Rheumatology, Rosalind Russell/Ephraim P Engleman Rheumatology Research Center, University of California San Francisco, San Francisco, California 94143, USA; 56 Division of Rheumatology and Clinical Immunology, University of Pittsburgh, Pittsburgh, Pennsylvania 15261, USA; 57 Department of Medicine, Rheumatology Unit, Karolinska Hospital and Karolinska Institutet, Solna, Stockholm 171 76, Sweden; 58 Robert S. Boas Center for Genomics and Human Genetics, Feinstein Institute for Medical Research, North Shore LIJ Health System, Manhasset, New York 11030, USA; 59 Merck Research Labs, Merck and Co, Boston, Massachusetts 02115, USA

## Abstract

Rheumatoid arthritis (RA) affects millions world-wide. While anti-TNF treatment is widely used to reduce disease progression, treatment fails in ∼one-third of patients. No biomarker currently exists that identifies non-responders before treatment. A rigorous community-based assessment of the utility of SNP data for predicting anti-TNF treatment efficacy in RA patients was performed in the context of a DREAM Challenge (http://www.synapse.org/RA_Challenge). An open challenge framework enabled the comparative evaluation of predictions developed by 73 research groups using the most comprehensive available data and covering a wide range of state-of-the-art modelling methodologies. Despite a significant genetic heritability estimate of treatment non-response trait (*h*^2^=0.18, *P* value=0.02), no significant genetic contribution to prediction accuracy is observed. Results formally confirm the expectations of the rheumatology community that SNP information does not significantly improve predictive performance relative to standard clinical traits, thereby justifying a refocusing of future efforts on collection of other data.

## Abstract (web summary)

Rheumatoid arthritis patients respond differently to anti-TNF treatment. Using community-based challenge, the authors show that currently available data does not reveal meaningful genetic predictors of response to anti-TNF therapy, thus confirming clinical observations.

## Graphical abstract

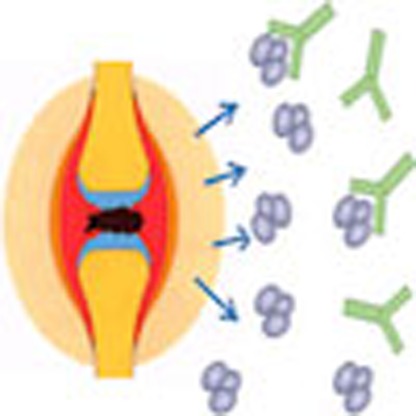

Rheumatoid arthritis (RA) is a chronic inflammatory autoimmune disorder affecting synovial joints, which often leads to organ system disorders and increased mortality. It is the most common autoimmune disorder, affecting ∼1% of the population worldwide^1^. RA is treated in part with disease-modifying anti-rheumatic drugs, including those that block the inflammatory cytokine, tumour necrosis factor-α (anti-TNF therapy). While anti-TNF treatment is effective in reducing disease progression, response is variable with nearly one-third of RA patients failing to enter clinical remission^2^,^3^,^4^. No substantive methodology exists that can be used to *a priori* identify anti-TNF non-responders^5^. Technological advances in DNA genotyping and sequencing have afforded the opportunity to assess the contribution of genetic variation to heterogeneity in anti-TNF response to therapy. Evidence from association analyses^6^,^7^ and theoretical heritability estimates suggested that algorithms focusing on genetic variation may be predictive of non-response. Genetic biomarkers provide a compelling opportunity to perform simple tests with high-potential impact on clinical care. Although genetic information has not been found to provide clinically relevant predictions in many cases^8^,^9^,^10^, the high-potential impact of successful genetic biomarkers and their potential to provide biological insights continues to inspire research inquiries in many fields including anti-TNF response. To this aim, we perform a community-based empirical assessment of the contribution of common single-nucleotide polymorphism (SNP) data to predictions of anti-TNF treatment response in RA patients to formally assess their utility for clinical application. Using the most comprehensive data set currently available, which we demonstrate is suitably powered to develop clinically actionable predictors, we draw on the expertise of hundreds of researchers world-wide, who collectively submit thousands of models predicting anti-TNF response. While the researchers are able to build predictive models that perform significantly better than random, formal evaluation from the best-performing teams show that common SNP variants do not meaningfully contribute to model performance within this study.

## Results

### Study design and challenge parameters

This study was performed as an open analytical challenge using the DREAM framework^11^,^12^,^13^,^14^ (DREAM Challenges website; www.dreamchallenges.org) as a mechanism to test predictions developed across a variety of state-of-the-art methodologies. In this manner, we were able to evaluate the accuracy of predictive models developed by dozens of research groups across a wide spectrum of modelling approaches. Challenge participants were provided with SNP data collected on 2,706 anti-TNF-treated RA patients^6^ (Supplementary Table 1) with which to develop predictive models of disease-modulating treatment response where treatment efficacy was measured using (a) the absolute change in disease activity score in 28 joints^15^ (DAS28) following 3–6 months of anti-TNF treatment and (b) categorical non-response as defined by EULAR-response criteria^16^. EULAR response is calculated based on the pre- and post-treatment DAS28 and is widely used in clinical research and practice. Models were evaluated based on the predictive accuracy in a held-out test data set containing 591 anti-TNF-treated RA patients from a separate cohort (Supplementary Table 1). This represents the most comprehensive set of data available to address this question.

### Statistical assessment of study power

The feasibility of developing SNP-based predictive models given this collection of data was determined in three steps. First, the genetic contribution to overall variance in treatment efficacy was estimated. Significant SNP-heritability estimates were identified via variance component modelling^17^,^18^ of common SNPs (minor allele frequency (MAF)≥0.01) within the primary cohort consisting of 2,706 patients from 13 studies^6^ (SNP-*h*^2^=0.18, *P* value=0.02, Table 1). Heritability estimates were strongest in the subset of patients treated with anti-TNF monoclonal antibodies (MABs) relative to those treated with the circulating biologic, enteracept (Table 1). These heritability estimates are similar to those reported for other treatment response traits^19^ and of sufficient effect size to consider the use of predictive modelling methods to identify polygenic predictors of anti-TNF treatment efficacy^20^,^21^. As the second step, the proportion of SNP heritability that must be represented in a predictive model to provide a clinically actionable predictor was estimated. Although the definition of an actionable predictor is highly dependent on clinical context, we set a lower bound, as defined by the area under the receiver operating characteristic curve (AUROC), of 0.75. Maximum AUROC achievable was calculated for the primary endpoint used in the challenge, categorical non-response based on the EULAR criteria and assuming the above estimated SNP heritability. Given these assumptions, the achievable AUROC was estimated across a set of predictive models as a function of the percent heritability explained by the model^22^. These estimates indicated that clinically actionable predictors would require predictive models to explain at least 55% of the heritable component of treatment efficacy (Supplementary Table 2). As the third and final step, statistical power to build such predictive models was estimated given these data. Statistical power was defined as the expected percent heritability that can be explained by a given model. This measure was computed over a range of models representing common risk variants (MAF≥0.01). In each case, models were consistent with the estimated SNP-*h*^2^ and non-response prevalence rate calculated within these data and assumed a fixed number of loci of equal effect^8^ (Supplementary Fig. 1a). Because previous analyses of these data did not reveal strongly associated individual loci, and ultimately many teams limit multiple testing through biologically informed feature selection^23^ (see Supplementary Table 3), this analysis was performed across a series of significance thresholds, ranging from that appropriate for genome-wide association (5e−8) to that appropriate for testing 100 independent loci (5e−4). These estimates indicated that this study was powered to develop clinically actionable predictive models in the case where the observed SNP heritability was explained by tens of risk loci. In this range, dimensionality reduction through literature or database curation could extract information even when strong, genome-wide significant associations are not observed. More sophisticated simulations using similar sample and disease characteristics suggest that the power estimates presented here may be conservative^8^. Despite smaller sample sizes, we estimate slightly increased power to build clinically meaningful predictors in the subset of patients treated with anti-TNF MABs. While the number of true underlying loci that contribute to genetic risk for anti-TNF response is unknown, an assumption of tens of loci is supported by observations of small numbers of loci associated with other treatment response traits^24^,^25^,^26^ and has the added advantage of approaching the number of loci that are practical to include on a clinical diagnostic panel. It should also be noted that our power calculations require a fixed level of statistical significance be achieved for model inclusion of SNP predictors, however, the inclusion requirements for many machine learning approaches are not as strict and, as such, these methods may be better powered^23^.

### Open challenge study design

**Figure 1:**
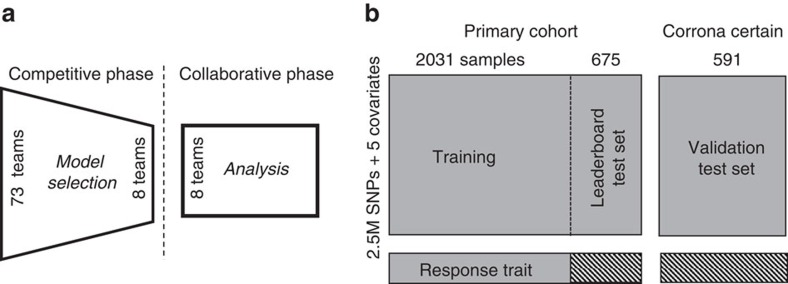
Challenge schematic. (**a**) This analysis was performed in two phases. In the Competitive phase, an open competition was performed to formally evaluate and identify the best models in the world to address this research question. In all, 73 teams representing 242 registered participants joined the challenge. Organizers evaluated model performance for test set predictions submitted by 17 teams. The 8 best-performing teams were invited to join the collaborative phase. In this phase, a collectively designed experiment was developed, in which each team independently performed analyses and challenge organizers performed a combined analysis. (**b**) Two data sets were used in the analysis: the Discovery cohort and the CORRONA CERTAIN study. Participants were provided with 2.5M imputed SNP genotypes+5 covariates from two cohorts and with the response trait for 2,031 individuals in the Discovery cohort (‘Training Set'). At the completion of the 16-week training period, participants were required to submit a final submission containing predictions of response traits in a completely independent data set, the CORRONA CERTAIN study (‘Validation Test Set').

The open challenge was designed to assess genetic contribution to prediction of anti-TNF response in RA patients using whole-genome SNP data derived from anti-TNF-treated RA patients (Fig. 1a, Supplementary Table 1)^6^,^27^. The question of anti-TNF treatment response was addressed in two ways. The primary endpoint used in the challenge was the classification of response to anti-TNF therapy as defined by EULAR-response criteria^16^ (Classification subchallenge). Participants were also invited to directly predict ΔDAS28 as a continuous measure (Quantitative subchallenge). In total, 242 individuals representing 30 countries and 4 continents registered to participate in this challenge. Challenge participants were invited to train models using a data set containing whole-genome SNP data, age, sex, anti-TNF therapy, concomitant methotrexate treatment and baseline DAS28 in a subset of 2,031 individuals (Fig. 1b, Supplementary Table 1 and see the ‘Methods' section)^6^. SNP data were provided as imputed (HapMap phase 2) genotype probabilities and dosages, as well as directly assayed variants for participant use.

Participants were provided with a leaderboard with real-time feedback, which evaluated the performance of their predictions in the remaining 675 individuals. To reduce the potential for overfitting or reverse-engineering of treatment outcomes from the leaderboard, each team was limited to 100 leaderboard submissions. Over the course of the 16-week training period, 73 teams submitted a total of 4,874 predictions for evaluation on the leaderboard data. Upon completion of the training period, teams were allowed up to two final submissions per subchallenge and final evaluation of algorithms was performed relative to a separate test data set consisting of data collected from 591 RA patients in the CORRONA CERTAIN^27^ study. Comparison with an independent, blinded test data set reduced the contribution to estimated accuracy of overfitting to the training data set, as indicated by comparing predictive performance between leaderboard and test data predictions for both the area under precision-recall curves (AUPRs) and AUROCs (Supplementary Fig. 2). Anti-TNF non-response differed slightly between the training and test data sets (21.7 and 35.7%, respectively), likely due to differences in inclusion criteria in the two cohorts, although demographic data were similar between the two (Supplementary Table 1). Similar methods were used to quality control (QC) and impute genotypes in both cohorts (see the ‘Methods' section for details). Participants remained blinded to outcomes from both the leaderboard and test data sets throughout the experiment. Harmonized data from all cohorts are publicly available as a resource for use by the research community (doi:10.7303/syn3280809).

### Performance across predictive modelling methodologies

**Figure 2:**
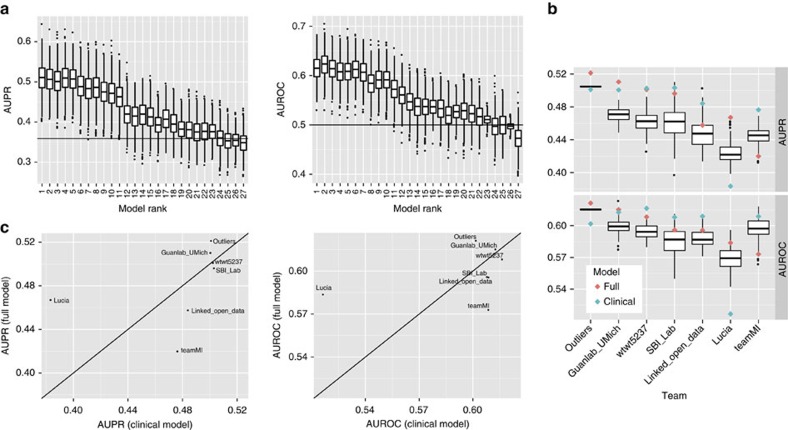
Model performance. Competitive Phase: (**a**) Bootstrap distributions for each of the 27 models submitted to the classification subchallenge ordered by overall rank. The top 11 models were significantly better than random at Bonferroni-corrected *P* value<0.05. Collaborative Phase: (**b**) Distributions of the models built with randomly sampled SNPs, by team, along with scores for their full model, containing data-driven SNP, as well as clinical variable selection, (pink) and clinical model, which contains clinical variables but excludes SNP data (blue). For 5 of 7 teams, the full models are nominally significantly better relative to the random SNP models for AUPR, AUROC or both (enrichment *P* value 4.2e−5). (**c**) AUPR and AUROC of each collaborative phase team's full model, containing SNP and clinical predictors, versus their clinical model, which does not consider SNP predictors. There was no significant difference in either metric between models developed in the presence or absence of genetic information (paired *t*-test *P* value=0.85, 0.82, for AUPR and AUROC, respectively).

For the classification subchallenge, 27 final submissions were received from 15 teams and these were scored using both AUROC and AUPR. Overall rank for each submission was determined as the average of the AUROC rank and the AUPR rank among all valid submissions. AUROC and AUPR were interpolated in the case of binary classifications or in the case of tied predictions^28^. Of 27 submissions, 11 performed significantly better than random for both AUPR and AUROC after Bonferroni correction for multiple submissions. The AUPR of all submissions ranged from 0.345 to 0.510 (null expectation 0.359), and the AUROC ranged from 0.471 to 0.624 (null expectation 0.5). Using bootstrap analysis of submission ranks (Fig. 2a), we determined that the top two submissions performed robustly better than all remaining solutions (Wilcoxon signed-rank test of bootstraps *P* value=5e−34 and 1e−66, relative to the third ranked submission, respectively) but were not distinct from one another (*P* value=0.44). These submissions had AUPR of 0.5099 and 0.5071 and AUROC of 0.6152 and 0.6237, respectively. Both of the top-performing submissions were generated using Gaussian Process Regression (GPR)^29^ models. Team Guanlab selected SNP predictors based on analysis of the training data and previous analyses described in the literature and, using those features, applied a GPR model to predict non-response classification directly. Team SBI_Lab selected SNP predictors based entirely on analysis of the training data, applied a GPR model to predict ΔDAS28, and then refactored these predictions into classification weights. The code and provenance for the winning algorithms have been catalogued and made available for reuse through the challenge website (see ‘Team Guanlab' and ‘Team SBI_Lab' in the Supplementary Notes for more details).

For the quantitative subchallenge, 28 final models from 17 teams were received and performance was evaluated based on the correlation between predicted and observed ΔDAS28 (observed range: *r*=0.393 to −0.356). Of these, 18 submissions performed significantly better than random at a Bonferroni-corrected *P* value threshold of 0.05 (*r*=0.393 to 0.208). The top-performing submission, provided by Team Guanlab, was robustly better than all remaining solutions (Wilcoxon's signed-rank test of bootstraps *P* value=2e−32 relative to the second ranked submission, Supplementary Fig. 3) and used a similar GPR model to predict ΔDAS28 as described above (see ‘Team Guanlab' in the Supplementary Notes for more details).

### Genetic contribution to model performance

Following the completion of the open challenge, the eight teams with the best predictive performances (seven from each subchallenge) were invited to join challenge organizers to perform a formalized evaluation of the contribution of genetic information to model performance across the solution space captured by these diverse methods. Challenge participants and organizers worked together in a collaborative manner to design and implement analyses to address this question. To ensure this analysis was performed across the best possible methods, teams were invited to continue to refine their individual algorithms based on the information shared across teams. Crosstalk was promoted through a webinar series for the discussion of methodological considerations between challenge teams, organizers and external experts. In addition, the eight teams were divided into three groups where intra-group discussions were encouraged. In general, we observed that teams altered their strategies regarding knowledge mining and feature selection in response to these efforts, but did not alter machine learning algorithms (Supplementary Table 3). Collaborative Phase predictions did not perform significantly better than Open Challenge predictions among collaborative phase participants (−0.017 ΔAUPR and −0.011 ΔAUROC for classification subchallenge, *P* value=0.270 and 0.265, respectively), further supporting the hypothesis that the overall genetic contribution to predictive performance was negligible.

To explicitly test the ability of modelling techniques to detect weak genetic contribution, we first examined the contribution of feature selection to model performance. Most teams had used a combination of knowledge-based and data-driven evidence to perform dimensionality reduction in their model development. To approximate the null distribution of the genetic models, each of the 8 teams trained 100 models using an equivalent number of randomly sampled SNPs relative to their best-performing model^30^. For 5 of 7 classification algorithms, models using knowledge-mined SNP selection significantly outperformed models using random SNPs for AUPR, AUROC or both at a nominal *P* value<0.05 (one-sided Kolmogorov–Smirnov test for enrichment of *P* values versus uniform *P* value=4.2e−05; Fig. 2b). Although there is uncertainty in the estimates of individual-algorithm enrichment due to the relatively small numbers of resamplings that were performed, a sensitivity analysis indicated that this experiment-wide significant enrichment was robust to these uncertainties (enrichment *P* value=0.002 at the 99.87% upper confidence bound of estimated *P* values). This suggested that for these models there was a non-zero contribution of genetic information to treatment effect. We next performed a pairwise comparison to directly assess the practical contribution of genetic information to model performance. Each team developed a model built in the absence of genetic information (clinical model) against which we compared their best model incorporating SNP data (full model). Clinical and demographic covariates were available for incorporation in both cases. Pairwise comparison across models demonstrated no statistical difference (paired *t*-test *P* value=0.85, 0.82, for classification AUPR and AUROC, respectively, and *P* value=0.65 for continuous prediction correlation; Fig. 2c, Supplementary Fig. 4), indicating that the contribution of SNP data to the prediction of treatment effect was not of sufficient magnitude to provide a detectable contribution to overall predictive performance. In further support of this conclusion, we note that the top-performing regression-based model by Team Outliers did not include any contribution from genetic information—genetic information was provided as an input but regularized out as part of the parameter selection process (see Supplementary Information). Despite the fact that heritability estimates were highest in the MAB therapies (adalimumab and infliximab), and that the most effective approaches explicitly modelled drug-specific genetic signal, there was no evidence that the genetic information contributed substantially for any drug-specific subset of the data (Bonferroni-corrected paired *t*-test *P* value for classification AUPR=1.0, 1.0, 1.0, 0.29, 1.0 for adalimumab, certolizumab, etanercept, infliximab and the combined set of all MAB therapies, respectively, and for AUROC=1.0, 1.0, 1.0, 0.59, 1.0, respectively).

The use of a diverse set of methodological approaches across teams provided the opportunity to test whether an aggregate of the individual approaches that leveraged their diversity/complementarity may boost the overall genetic contribution to predictions. Specifically, ensemble models were learned from individual predictions submitted to the classification subchallenge using a supervised approach^31^. These models were trained using leave-one-out cross-validated predictions generated on the original training set using the individual methods, and, as with individual submissions, analysed in a blinded manner using the test data. Two ensemble models were developed for the classification subchallenge using the stacking method^32^,^33^, one built using team predictions submitted during the open challenge phase (AUPR=0.5228, AUROC=0.622) and the second built using team predictions submitted during the collaborative phase (AUPR=0.5209, AUROC=0.6168). Performance of these supervised ensemble models was compared with performance of the individual team model with the best overall performance—the model submitted in the collaborative phase by Team Outliers that did not contain any genetic information (Supplementary Fig. 5). The ensemble models performed incrementally better than this model (differences=0.005 and 0.0006 and boostrap *P* values=0.32 and 0.46 for AUPR and AUROC, respectively). This indicates that even ensembles that leverage complementary information among the individual predictions could not boost the ability to robustly predict anti-TNF response using genetic information.

## Discussion

The RA Responder DREAM challenge performed a community-based open assessment of the contribution of SNP genotypes to predict disease-modulating response to anti-TNF treatment in RA patients, and found that SNPs did not meaningfully contribute to the prediction of treatment response above the available clinical predictors (sex, age, anti-TNF drug name and methotrexate use). Given the negative nature of the findings in this report, it is important to clearly frame these findings within the constraints of the problem that was addressed. This study was designed to assess the ability to develop clinically actionable predictors using common SNP variants in the case where the genetic contribution to treatment efficacy is represented by tens of loci. Thorough analysis by dozens of researchers has shown that current predictive algorithms, as well as their ensembles, are not able to produce such predictors despite the estimation of a significant heritability for this trait. In fact, these researchers were not able to detect any genetic contribution to predictions, even in the subset of data for which heritability and power are predicted to be the highest. This may reflect the complex nature of genetic contribution across loci, the absence of individual, strongly associated common variants, or the presence of non-genetic sources of heterogeneity across individuals^8^,^9^,^10^. These findings do not provide information about the ability to use genetic data for predictive modelling of anti-TNF treatment efficacy in other cases such as when: (1) the true number of risk loci is on the order of hundreds, or (2) the heritability is better explained by variants not assayed or tagged by variants in this study, including rare variants or CNVs. Given the sample sizes required to identify loci when the number of risk loci is on the order of hundreds (Supplementary Fig. 1b), and the general challenge in explaining estimated heritability in complex traits even with large cohorts^34^,^35^,^36^,^37^,^38^, this does suggest that future efforts may be better spent in identifying biomarkers based on data modalities that better encapsulate both genetic and non-genetic contributions to treatment efficacy.

Although these genetic data did not provide a meaningful contribution to the predictions in this study, the methods used in this analysis were able to leverage the small set of available clinical features to develop a prediction that performed significantly better than random. These results suggest that future research efforts focused on the incorporation of a richer set of clinical information—including seropositivity, treatment compliance and disease duration—may provide opportunity to leverage these methods in clinically meaningful ways. In addition, the identification of data modalities that are more effective than genetics in capturing heterogeneity in RA disease progression—whether clinical, molecular or other—may also improve predictive performance.

This study demonstrates that a formalized evaluation of a scientific question across a wide solution space can be effectively accomplished by combining resources—data and methodologies—across an open community of interested researchers. In research areas of high-potential impact but uncertain likelihood of success, such as described here, this community-wide approach provides an opportunity to build consensus regarding research outcomes to guide future efforts within that field. In this context, positive outcomes can highlight a rich strategy for future enquiry, while negative results can provide strong evidence in support of adjusting future paths of scientific exploration. Since the evidence that a task is implausible mounts with the number of failed attempts at solving it, making the case of implausibility requires the active contribution of multiple research groups. In this study, we demonstrate that formalized evaluation across a community of researchers provided a rapid mechanism for transparent assessment of current capabilities to assess the contribution of genetic information to prediction of anti-TNF response in RA patients.

## Methods

### Data sets

Two separate data sets were provided to participants to train and test the predictive models, respectively (Supplementary Table 1). In the case of the test data, only predictor variables were released, and the teams remained blinded to the response variables. The training data consisted of a previously published collection of anti-TNF-treated patients (*n*=2,706) of European ancestry, compiled from 13 collections^6^, of which the response variables from 675 patients were held-out as a leaderboard test set. All patients met 1987 ACR criteria for RA, or were diagnosed by a board-certified rheumatologist and were required to have at least moderate DAS28 (ref. 15 at baseline (DAS28>3.2). Available clinical and demographic data included DAS28 at baseline and at least one time point after treatment, sex, age, anti-TNF drug name and methotrexate use. Follow-up DAS28 was measured 3–12 months after initiating anti-TNF therapy, though precise duration of treatment was not available. Genotypes for each sample were analysed for quality control (QC) for sample and marker missingness, Hardy–Weinberg disequlibrium, relatedness and population outliers, and imputed to HapMap Phase 2 (release 22) as previously described^6^. We note that although this data set does not represent the full spectrum of patient information that may be utilized within a clinical setting to inform treatment—including synovial tissue and novel soluble biomarkers like MRP8/14 levels^4^,^39^,^40^, it did present sufficient data to explicitly assess the contribution of genetics to prediction.

The final test set was derived from a subset of patients enroled in the CORRONA CERTAIN study^27^. CERTAIN is a prospective, non-randomized comparative effectiveness study of 2,804 adult patients with RA, having at least moderate disease activity defined by a clinical disease activity index score >10 who are starting or switching biologic agents. DAS28 was provided at baseline and 3-month follow-up. At the time of challenge launch, 723 subjects had initiated anti-TNF therapy and had a 3-month follow-up visit. Of these patients, 57.4% were previously naive to biologics. Genotypes were generated on the Illumina Infinium HumanCoreExome array and imputed to HapMap Phase 2 (release 22) using IMPUTE2 (ref. 41). Before imputation, genotype QC included filtering individuals with >5% missing data, and filtering SNPs with >1% missing data, MAF<1% and *χ*^2^-test of Hardy-Weinberg equilibrium *p*_HWE_<10-5. Sex was inferred based on the X-chromosome genotypes using PLINK^42^, and all samples matched with respect to reported sex. One parent-offspring relationship was identified in the data, but was kept in the test set. While data for all 723 were released to participants, 93 patients were excluded for the purposes of scoring because their genotyping data were not consistent with European ancestry as inferred by EIGENSTRAT^43^. In addition, a subset of patients in the test data set were treated with anti-TNF drugs that were not represented in the training data set: golimumab and certolizumab. The 39 patients receiving golimumab were excluded because this drug was not represented in the training data and predictions showed that participants were unable to successfully predict response in these subjects. In contrast, prediction in certolizumab-treated patients was similar to prediction in the remaining three drugs and so these data were included in the final test set.

Two ancillary data sets were made available for participant use. The first measured TNFα protein level in HapMap cell lines^44^. The second included blood RNA-seq data and genotypes for 60 RA patients from the Arthritis Foundation-sponsored Arthritis Internet Registry, 30 of whom displayed high inflammatory levels and 30 of whom displayed low inflammatory levels. Inflammatory levels were assessed using blood concentrations of C-reactive protein (CRP), and elevated disease was defined as CRP>0.8 mg dl^−1^, while low disease activity was defined as CRP<0.1 mg dl^−1^. In addition to CRP levels, rheumatoid factor antibody levels and cyclic citrullinated peptide levels were also assayed. Genotypes were assayed on the Illumina HumanOmniExpressExome array.

### Power calculations

For combinations of a range of risk allele frequency, *P*=(0.01, 0.02, 0.03, …, 0.99), and relative risk, *λ*=(1.1, 1.11, 1.12, …, 2.4), we computed the number, *n*, of such loci required to explain a heritability of 0.18, as estimated for this trait, (equation (3) in the study by Wray *et al.*^8^), and the power assuming a multiplicative model using the GeneticsDesign Bioconductor package^45^, given a trait prevalence, *K*=0.217, as estimated from the discovery cohort. The expected heritability explained was estimated as the median power over all combinations of *p* and *λ* for which *n* rounded to a given value.

The AUROC corresponding to various proportions of heritability explained was computed using equation (3) in the study by Wray *et al.*^22^ after converting our estimated heritability to the liability scale. In addition, we estimated the proportion of the variance explained by clinical variables using the AUROC for the best clinical model from the collaborative phase (equation (4) in the study by Wray *et al.*^22^) and computed the AUROC corresponding to various proportions of heritability explained assuming independence between the clinical and genetic components.

### Scoring methods

For the classification subchallenge, teams were asked to submit an ordered list of patients ranked according to the predicted response to therapy. Special treatment was given to the computation of the curve statistics when the order was ambiguous such as in the case in the case of ties or binary predictions, in which case an average across all possible consistent solutions was used^28^. The average of the rank of the AUPR and AUROC was used to rank solutions.

For the quantitative subchallenge, teams were asked to submit predicted ΔDAS28, and the Pearson's correlation between the predicted and actual ΔDAS28 was used to score submissions.

### Competitive phase of the challenge

The challenge was open to all individuals who agreed to the DREAM terms of use and obtained access to the challenge data by certifying their compliance with the data terms of use. The training and ancillary data were released for use on 10 February 2014. The leaderboards opened on 5 March, at which time participants were able to test their models in real-time against a held-out portion of the training data set. The prediction variables of the test data set were released to participants on 8 May and submission queues for final submissions were open between 21 May and 4 June. Only the final two submissions per team per subchallenge were scored. Participants who did not have enough computational resources in their home institutions were offered the option to use an IBM z-Enterprise cloud, with two virtual machines running Linux servers, one with 20 processors, 242 GB memory, 9 TB storage space and the other with 12 processors, 128 GB memory and 1 TB of storage space. Cloud users could access the Challenge data directly through the IBM system.

### Evaluation of submissions

Predictions were evaluated using two data sets: 675 individuals from the training cohort (leaderboard test set) and all individuals from the CORRONA CERTAIN data (final test set). In both cases, response variables were withheld from participants. Participants were allowed 100 submissions to the classification subchallenge leaderboard and unlimited submissions to the quantitative subchallenge leaderboard throughout the competitive phase of the competition, and were provided near-instant results. Participants were allowed two final submissions per subchallenge and scores were revealed after the submission deadline. A permutation test was used to assess whether the classifications or ΔDAS28 quantitative predictions were better than expected at random using a one-sided *P* value. To assess the robustness of the relative ranking of predictions, 1,000 bootstraps were performed by sampling subjects with replacement. Within each bootstrap iteration, evaluation scores were computed for each submission, along with the within-iteration rank. A prediction was deemed ‘robustly' better than another if the Wilcoxon's signed-rank test of the 1000 bootstrap iteration estimates was significant with *P* value<0.05. While this is not the same as strict statistical significance, it was the criteria we used to differentiate models given the relatively small improvements from one to another.

### Development and scoring in the collaborative phase

One of the aims of DREAM Challenges is to foster collaborative research. As such, the collaborative phase was designed to foster cooperation between the best-performing teams in the competitive phase. Teams came together to develop research questions and analytical strategies to answer specific questions related to the ability to predict non-response to anti-TNF treatment. Each team submitted a number of classifications/predictions and/or sets of classifications/predictions that were designed to be able to answer questions about the degree to which genetic data were contributing to the models, and the classifications were scored and analysed across teams by the challenge organizers. To compare across methods and approaches, we asked the collaborative phase participants to submit classifications/predictions using their own knowledge- and data-mined SNP lists, which they refined from the competitive phase after peer review from fellow participants. In addition, they were asked to submit a classification/prediction, which used only clinical predictors and did not include genetic predictors. We also asked the participants to submit 100 sets of classifications/predictions in which the SNPs used as potential predictors were randomly sampled from the genome and matched the number of SNPs in their genetic model. Eight teams participated in the collaborative phase, seven in each subchallenge. Ranked results for the genetic models are shown in Supplementary Fig. 5.

### Ensemble classifications

The goal of ensemble learning was to aggregate the classifications submitted by individual teams to the classification subchallenge, including 6 from the Competitive Phase and 7 from the Collaborative Phase, by effectively leveraging the consensus as well as diversity among these predictions. We focused on learning heterogeneous ensembles^31^, which are capable of aggregating classifications from a diverse set of potentially unrelated base classifiers, as is the case with the submissions to this subchallenge. Specifically, we followed the stacking methodology^32^,^33^, which involves learning a meta-classifier (second level predictor) on top of the base classifications. This methodology was applied to the training set classifications generated through a leave-one-out cross-validation procedure applied to the training set for the initial ensemble learning. To address the potential calibration issue in this task^46^, we investigated using the raw base classifications and the output of two other normalization procedures—*z*-score (mean=0, s.d.=1) and Scale0–1 (maximum=1, minimum=0)—applied to the raw base classifications. Next, sixteen different classification algorithms (Supplementary Table 4) were used to train ensemble models from each of the above normalized versions of the base classifications. The implementations of these algorithms were obtained from the Weka machine learning suite^47^, and their default parameters were used.

Supplementary Figure 6 shows the performance of different combinations of normalization and classification methods on the leaderboard test set in terms of (a) AUPR, (b) AUROC and (c) the overall rank. Several observations can be made from these results. First, the ensemble learned with normalization using *z*-score and subsequent learning of a Naive Bayes classifier that uses kernelized probability distribution functions^48^ produced the best aggregate performance on the leaderboard test set (AUROC=0.7569, AUPR=0.49), indicating the conditional independence of the base classifications and the non-normality of their underlying distributions. In general, normalization (either *z*-score or Scale0–1) improved the performance for 14, 14 and 13 of the 16 classifiers examined in terms of PR, ROC and overall rank, respectively, thus indicating the importance of effective calibration in such ensemble learning tasks. Of these, 10, 9 and 7 classifiers, including NaiveBayes_kdf, saw the best performance due to the use of *z*-score normalization, thus giving this normalization method an edge over Scale0–1.

On the basis of the conclusions above, we applied the ensemble model trained using *z*-score and NaiveBayes_kdf to the individual team classifications submitted for the CORRONA CERTAIN test set in the competitive and collaborative phases. The ensemble of the competitive phase (AUPR=0.5228, AUROC=0.622) performed better than each of the individual classifications and slightly better than the ensemble of the collaborative phase (AUPR=0.5209, AUROC=0.6168). However, these improvements were not statistically significant.

### Data availability

Data use within the scope of this challenge was performed with the approval of an internal review board for all data sets. All data used for the challenge are available through the Synapse repository (syn3280809, doi:10.7303/syn3280809).

## Additional information

**How to cite this article:** Sieberts, S. K. *et al.* Crowdsourced assessment of common genetic contribution to predicting anti-TNF treatment response in rheumatoid arthritis. *Nat. Commun.* 7:12460 doi: 10.1038/ncomms12460 (2016).

## Supplementary Material

Supplementary Information Supplementary Figures 1-6, Supplementary Tables 1-4, Supplementary Note 1 and Supplementary References

## Figures and Tables

**Table 1:** Heritability estimates within the Primary Cohort.

Gene list	*N* genes	Proportion of genome		SNP heritability (*P* value)	
		SNPs	Mb	All samples	Infliximab+Adalimumab
Whole genome	—	1	1	0.18 (0.02)	0.36 (0.005)
Drug metabolism*	215	0.07	0.10	0.05 (0.3)	0.04 (0.09)
Immune-related†	6,001	0.65	0.58	0.07 (0.2)	0.21 (0.01)
TNF/TNFR pathway‡	333	0.11	0.14	0.05 (0.04)	0.02 (0.3)
CD84 coexpression (ImmGen)	200	0.08	0.11	0 (0.5)	0 (0.5)

SNP, single-nucleotide polymorphism; TNF, tumour necrosis factor; TNFR, TNF-receptor.

^*^Affymetrix DMET chip SNPs.

^†^immport.niaid.nih.gov.

^‡^PPI and coexpressed genes (eQTLs).
