# Erratum: Crowdsourced assessment of common genetic contribution to predicting anti-TNF treatment response in rheumatoid arthritis

**DOI:** 10.1038/ncomms13205

**Published:** 2016-10-10

**Authors:** Solveig K. Sieberts, Fan Zhu, Javier García-García, Eli Stahl, Abhishek Pratap, Gaurav Pandey, Dimitrios Pappas, Daniel Aguilar, Bernat Anton, Jaume Bonet, Ridvan Eksi, Oriol Fornés, Emre Guney, Hongdong Li, Manuel Alejandro Marín, Bharat Panwar, Joan Planas-Iglesias, Daniel Poglayen, Jing Cui, Andre O. Falcao, Christine Suver, Bruce Hoff, Venkat S. K. Balagurusamy, Donna Dillenberger, Elias Chaibub Neto, Thea Norman, Tero Aittokallio, Muhammad Ammad-ud-din, Chloe-Agathe Azencott, Víctor Bellón, Valentina Boeva, Kerstin Bunte, Himanshu Chheda, Lu Cheng, Jukka Corander, Michel Dumontier, Anna Goldenberg, Peddinti Gopalacharyulu, Mohsen Hajiloo, Daniel Hidru, Alok Jaiswal, Samuel Kaski, Beyrem Khalfaoui, Suleiman Ali Khan, Eric R. Kramer, Pekka Marttinen, Aziz M. Mezlini, Bhuvan Molparia, Matti Pirinen, Janna Saarela, Matthias Samwald, Véronique Stoven, Hao Tang, Jing Tang, Ali Torkamani, Jean-Phillipe Vert, Bo Wang, Tao Wang, Krister Wennerberg, Nathan E. Wineinger, Guanghua Xiao, Yang Xie, Rae Yeung, Xiaowei Zhan, Cheng Zhao, Members of the Rheumatoid Arthritis Challenge Consortium, Manuel Calaza, Haitham Elmarakeby, Lenwood S. Heath, Quan Long, Jonathan D. Moore, Stephen Obol Opiyo, Richard S. Savage, Jun Zhu, Jeff Greenberg, Joel Kremer, Kaleb Michaud, Anne Barton, Marieke Coenen, Xavier Mariette, Corinne Miceli, Nancy Shadick, Michael Weinblatt, Niek de Vries, Paul P. Tak, Danielle Gerlag, Tom W. J. Huizinga, Fina Kurreeman, Cornelia F. Allaart, S. Louis Bridges Jr., Lindsey Criswell, Larry Moreland, Lars Klareskog, Saedis Saevarsdottir, Leonid Padyukov, Peter K. Gregersen, Stephen Friend, Robert Plenge, Gustavo Stolovitzky, Baldo Oliva, Yuanfang Guan, Lara M. Mangravite

Nature Communications 7: Article number: 1246010.1038/ncomms12460 (2016); Published: 08 23 2016; Updated: 10 10 2016.

The HTML version of this Article incorrectly duplicated the authors S. Louis Bridges, Lindsey Criswell, Larry Moreland, Lars Klareskog, Saedis Saevarsdottir, Leonid Padyukov, Peter K. Gregersen, Stephen Friend, Robert Plenge, Gustavo Stolovitzky, Baldo Oliva, Yuanfang Guan and Lara M. Mangravite in the author list. This has now been corrected in the HTML; the PDF version of the paper was correct from the time of publication.

