# Supplementary material for: Crowdsourced assessment of common genetic contribution to predicting anti-TNF treatment response in rheumatoid arthritis
Source: Nat Commun. 2016 Aug 23;7:12460. doi: 10.1038/ncomms12460 (PMC4996969; doi:10.1038/ncomms12460)
Supplement: Supplementary Information — Supplementary Figures 1-6, Supplementary Tables 1-4, Supplementary Note 1 and Supplementary References [file ncomms12460-s1.pdf]

Supplementary Figures

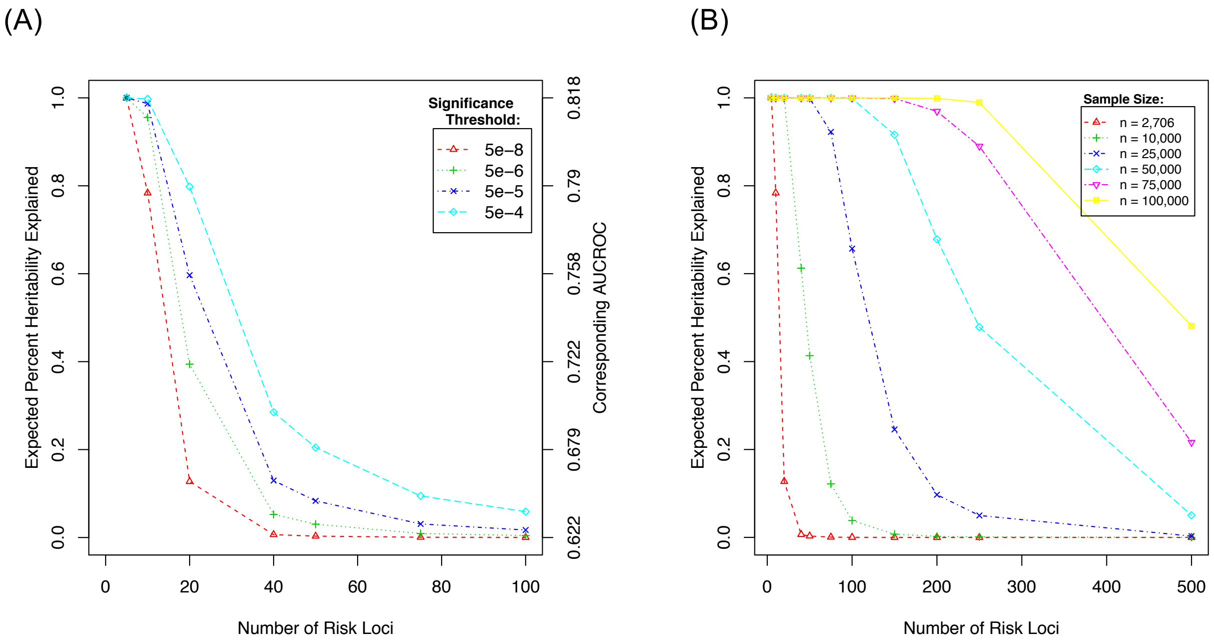

Supplementary Figure 1: **Power.** Expected percent heritability explained versus number of risk loci contributing to the genetic component: (A) for the discovery cohort at significance thresholds 5e-8 (genomewide significance), 5e-6 (correction for testing 10,000 independent loci), 5e-5 (correction for testing 1,000 independent loci) and 5e-4 (correction for testing 100 independent loci), and including expected AUROC corresponding to the percent heritability explained (y-axis, right), and (B) for various sample sizes assuming a genomewide significance cutoff of 5e-8 for inclusion in the model. Expected percent heritability explained is computed assuming population prevalence of 0.217 and heritability (h^2) of 0.18 as estimated from the discovery cohort.

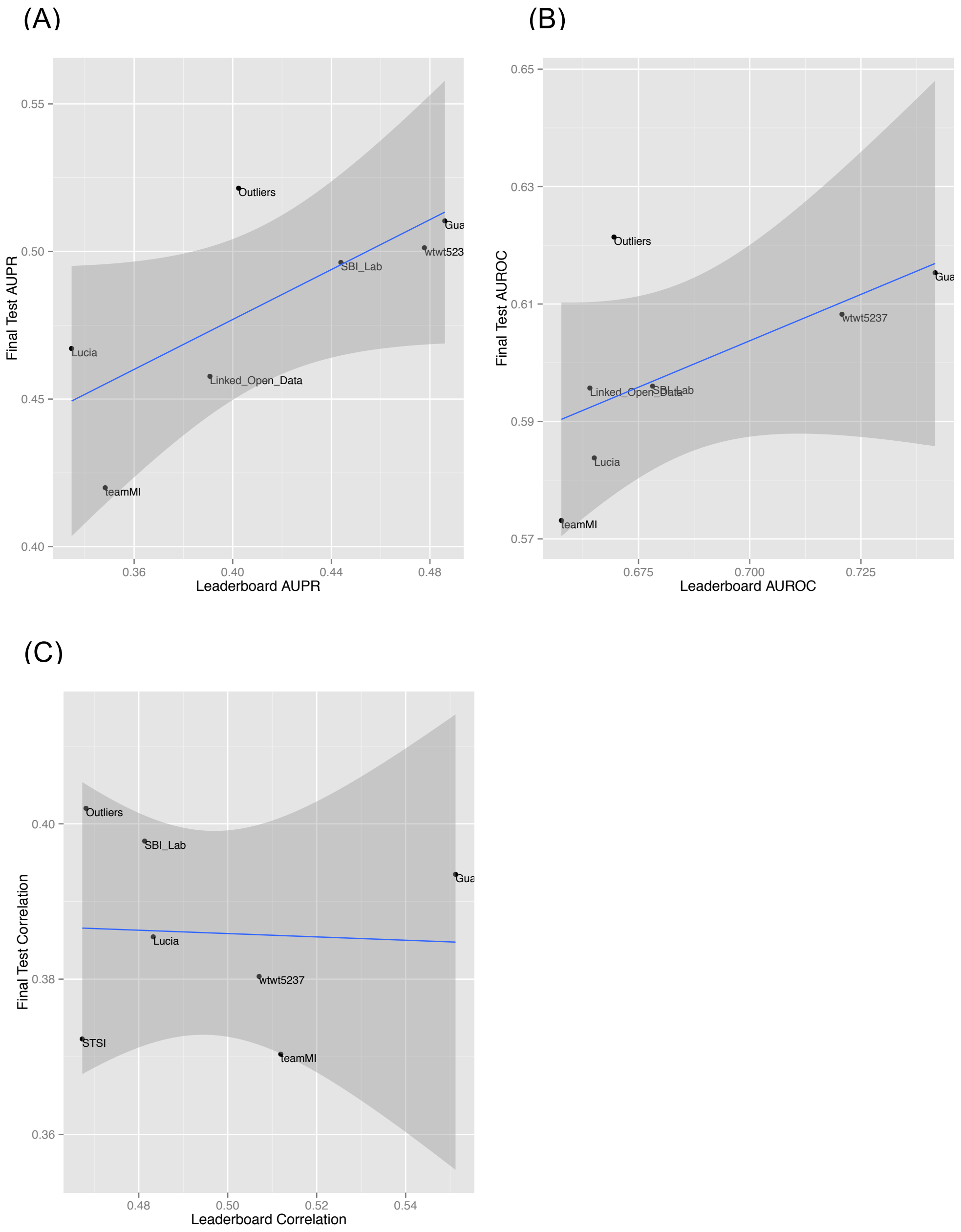

Supplementary Figure 2: **Final versus leaderboard performance.** Collaborative phase leaderboard score versus final submission score for AUPR, AUROC for the classification subchallenge, and correlation for the quantitative subchallenge with linear regression fit and 95% confidence region (shaded). While the two metrics for the classification subchallenge showed positive correlation (*r*= 0.71 and 0.60 for AUPR and AUROC, respectively) between the scores on the leaderboard data, which is a held-out portion of the training dataset, and the scores on the test data, the quantitative prediction subchallenge showed a negative correlation (*r*= -0.052) suggesting a tendency toward overfitting in that subchallenge.

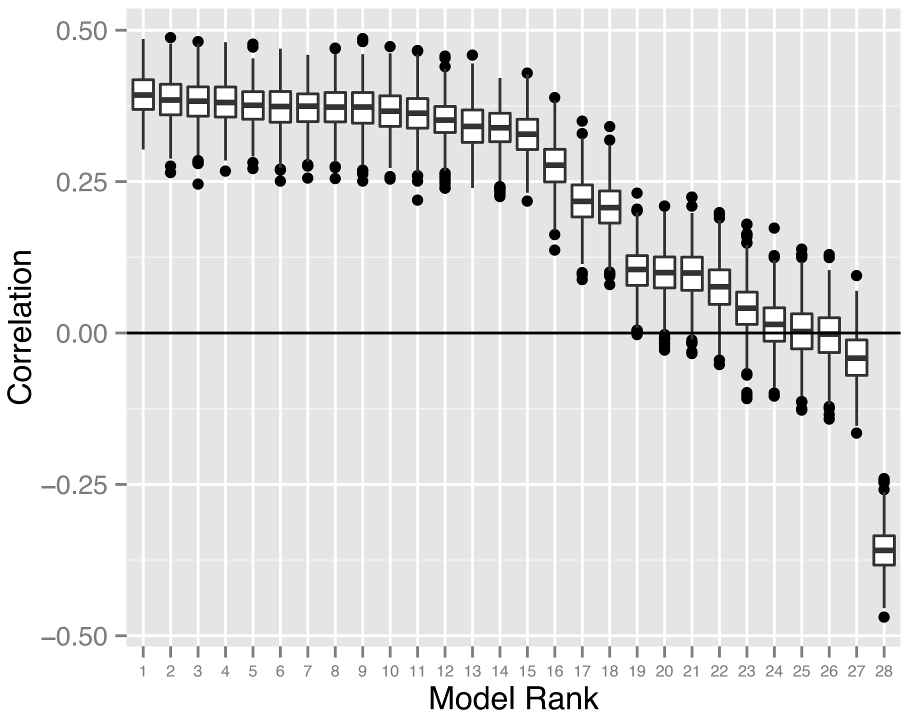

Supplementary Figure 3: **Competitive phase performance.** Bootstrap distributions of the 28 models submitted to the prediction subchallenge during the competitive phase, ordered by submission rank. The top 18 submissions performed better than random with Bonferroni corrected p-value < 0.05.

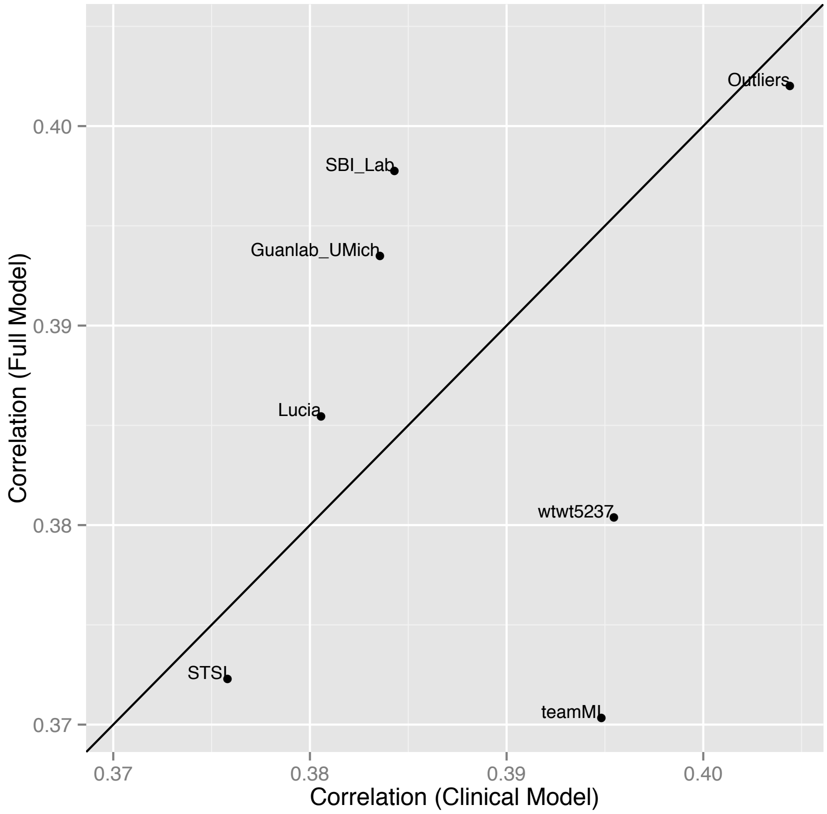

Supplementary Figure 4: **Full model versus clinical model performance.** Score (correlation with true values) of each team's collaborative phase full model, incorporating SNP and clinical data, versus their clinical model, which excludes SNP information, for the quantitative prediction subchallenge. There was no significant difference between full and clinical models (paired t-test *p*-value = 0.65).

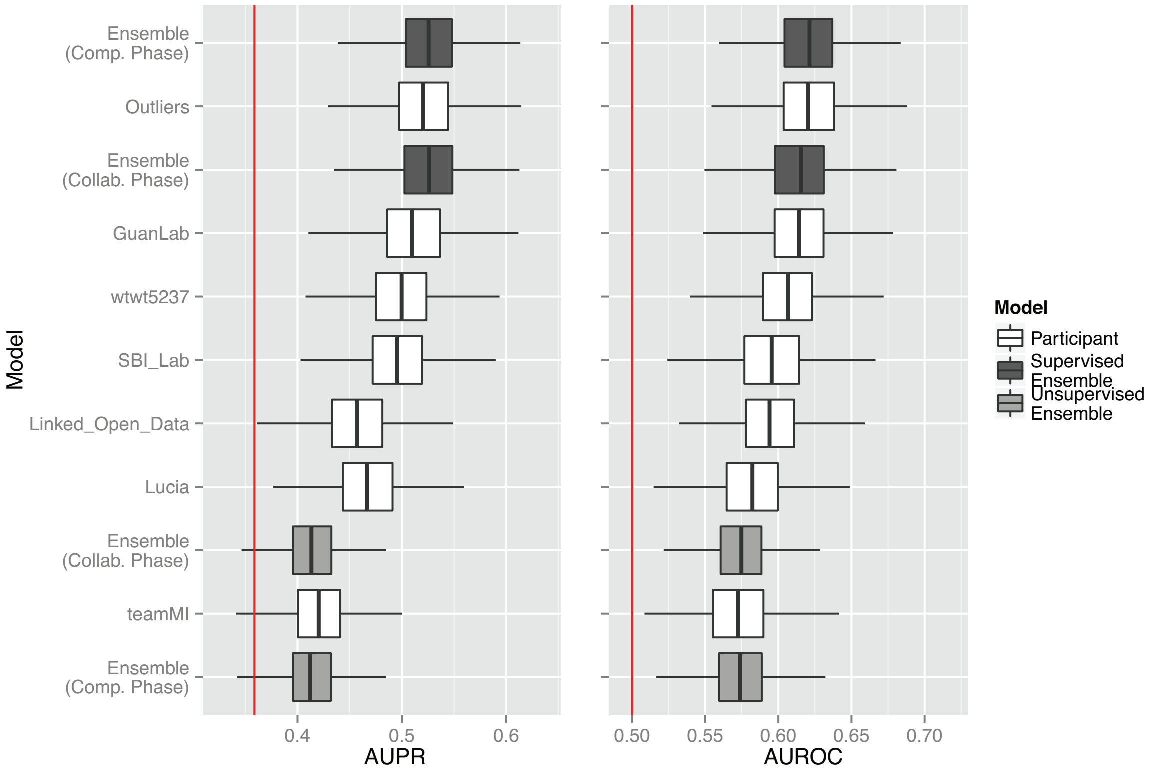

Supplementary Figure 5: **Collaborative phase results.** Bootstrap distributions of the team (participant) and ensemble models generated during the collaborative phase of the challenge, ordered by overall rank. While the supervised ensemble models showed general improvement of the individual team models, particularly when built using the competitive phase submissions, unsupervised ensemble models actually showed diminished scores relative to individual team models.

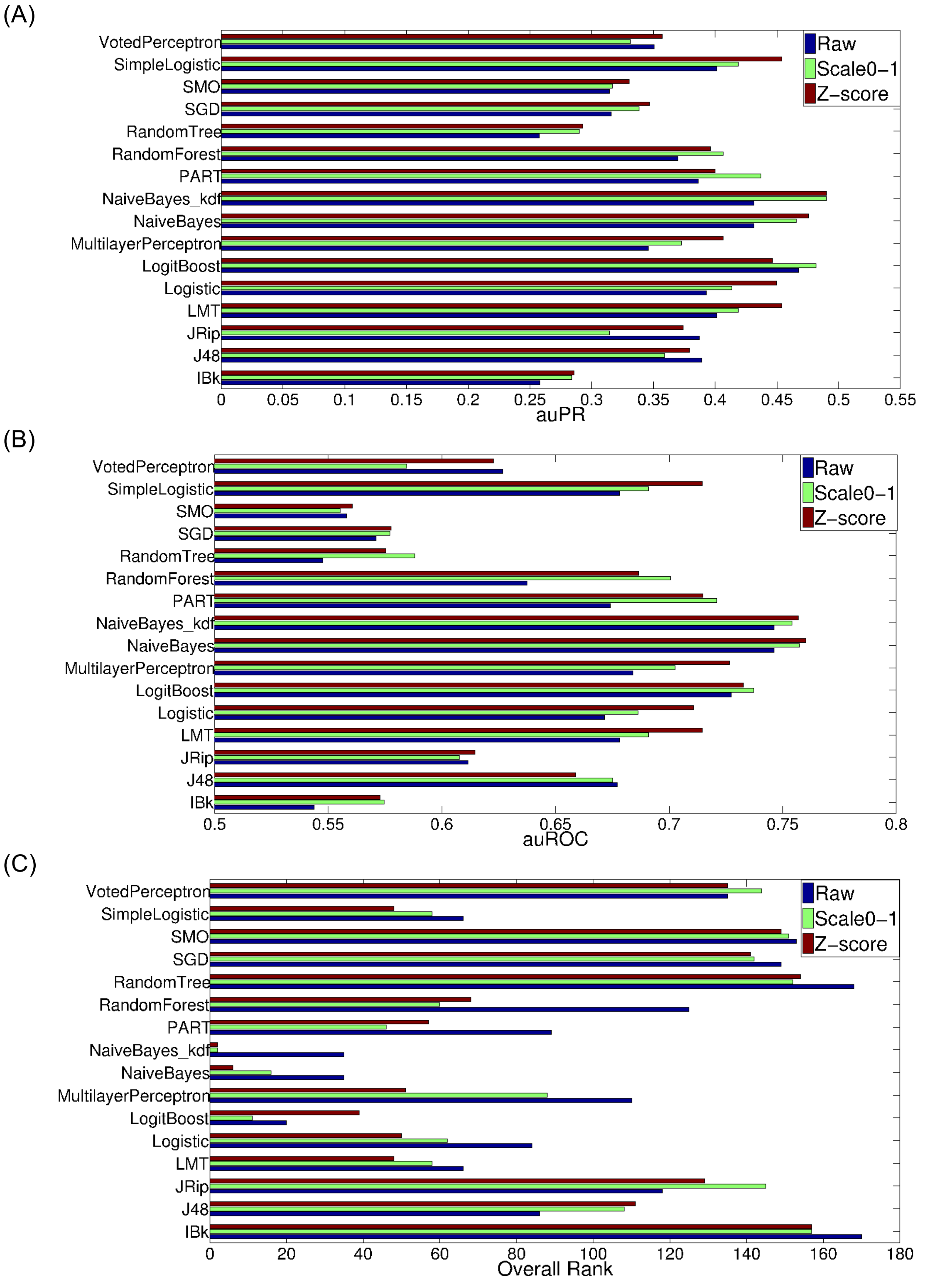

Supplementary Figure 6: **Ensemble method performance.** Relative performance of various combinations of prediction (Y-axis) and normalization methods (color-coded bars) in terms of (A) AUPR, (B) AUROC and (C) Overall rank.

Supplementary Tables

Supplementary Table 1: Cohort Characteristics

	**Primary Cohort**	**CERTAIN Cohort**
Patient numbers (%) by Treatment		
	Count (%)	Count (%)
Adalimumab	1071 (39.7)	210 (29.0)
Etanercept	733 (27.2)	179 (24.8)
Infliximab	893 (33.1)	177 (24.5)
Certolizumab	0 (0)	114 (15.8)
Golimumab	0 (0)	43 (5.9)
% Methotrexate Cotherapy	70.3	65.0
Patient numbers (%) by EULAR Response		
Good	994 (36.7)	243 (33.6)
Intermediate	1124 (41.5)	222 (30.7)
Non-responder	588 (21.7)	258 (35.7)
Clinical Characteristics		
% Female	73.9	77.7
Age Mean (SD)	54.8 (12.3)	55.4 (13.3)
Baseline DAS28 Mean (SD)	5.78 (1.22)	4.72 (1.08)
ΔDAS28 Mean (SD)	2.05 (1.48)	1.16 (1.26)

Supplementary Table 2: Maximum achievable area under the Receiver Operating Characteristic Curve (AUROC) as a function of percent heritability explained

Percent heritability explained	AUROC	
	Genetic component only	Genetic + clinical component*
0%	0.500	0.622
10%	0.592	0.653
20%	0.630	0.679
30%	0.659	0.702
40%	0.684	0.722
50%	0.706	0.741
60%	0.726	0.758
70%	0.745	0.775
80%	0.762	0.790
90%	0.778	0.805
100%	0.793	0.818

* Clinical component estimated from the best model fit using only clinical covariates in the collaborative phase of the analysis. See section ‘Genetic contribution to model performance’ in the main text for details. Clinical and genetic components are assumed to be independent.

Supplementary Table 3: Collaborative phase method summary

Team	Prediction Method	SNP Curation	Combined or by Drug	Drug	Number of SNPs
Guanlab_UMich	Gaussian Process Regression	Training Data and Literature	By Drug	Adalimumab	19
				Etanercept	14
				Infliximab	18
Linked Open Data	Gradient Booster Classifier	Training Data, RA GWAS, eQTL, pharmGKB	Combined	All	204
Lucia	Kernel Method	KEGG Pathways, Drugbank Targets	By Drug	All	21,684
Outliers	Hierarchical Regression	pharmGKB database, TNF eQTLs, TNF SNPs, TRAILR1 SNPSs TNFR1A SNPs	Combined	All	160
SBI_Lab	Gaussian Regression	Training Data	By Drug	Adalimumab	6
				Etanercept	22
				Infliximab	15
STSI	Tree-based Regression	RA GWAS, Training Data, eQTL, PPI, pharmGKB	Combined	All	372
TeamMI	Linear mixed effects model and multiple kernel learning	Training Data, RA GWAS	By Drug	Adalimumab	67
				Etanercept	79
				Infliximab	75
wtwt5237	Ensemble of Random Forest, SVM, Adaboost, LASSO, and PLS	GWAS Studies	Both	All	117

Supplementary Table 4: Classifiers (implemented in Weka[5]) that were used to learn stacking-based ensembles in this study

Classifier	Short description	Reference
VotedPerceptron	Voted Perceptron	[23]
SimpleLogistic	Linear combination of logistic model trees	[24,25]
SMO	Sequential minimal optimization algorithm for training an SVM	[26]
SGD	Stochastic gradient descent for learning linear models	[27]
RandomTree	Decision tree that considers K randomly chosen attributes at each node for splitting	[28]
RandomForest	Forest (collection) of random trees	[28]
PART	Rule set obtained from partial C4.5 decision trees	[29]
NaiveBayes	Naive Bayes classifier using normal distribution for continuous features	[30]
NaiveBayes_kdf	Naive Bayes classifier using kernel density estimator for continuous features	[30]
MultilayerPerceptron	Artificial feedforward neural network employing backpropagation for training	[31]
LogitBoost	Additive (boosting) logistic regression	[32]
Logistic	Multinomial logistic regression model with a ridge estimator	[33]
LMT	Logistic model tree	[24,25]
JRip	Propositional rule learner, Repeated Incremental Pruning to Produce Error Reduction (RIPPER)	[34]
J48	C4.5 decision tree	[35]
IBk	K-nearest neighbours classifier	[36]

Supplementary Note 1

Collaborative Phase Individual Team Methods

Solutions, write-ups and code for each collaborative phase team can be found in the Collaborative Phase Synapse project (https://www.synapse.org/#!Synapse:syn2509905).

Team Guanlab

Team Guanlab developed a Gaussian Process Regression (GPR)[1]-based model, or kriging, to predict drug response. The fundamental innovation of this method is that unlike other mainstream regression methods that take all training examples into consideration simultaneously, GPR predicts the treatment response for an individual patient by computing a weighted average of one to several most similar examples in the training set. Thus the weighting of different training examples is 'personalized' to each test example. Compared with other regression models, the advantage of GPR is that: instead of determining regression coefficients (describing how much each SNP could affect ΔDAS28 or Non-Response) for all of the selected SNPs, GPR calculates the similarities between test samples and training samples, and predict ΔDAS28 or Non-Response from only the most similar samples. Two types of features were used in GPR: genetic features and clinical features. For genetic features, we selected SNPs from both the training data and from literature. Based on our analysis of the data, after multiple testing correction, few SNPs can pass significance test using the training data alone. Thus, literature SNPs were sought, although many of them are not replicable in this training dataset and not necessarily replicable in future datasets. About half of the SNPs used in Team Guanlab's models were selected from published papers (i.e. those SNPs were claimed to be related to the risk of RA or anti-TNF treatment response). The SNPs selected from literature were tested using cross-validation in the training data, and the leaderboard, resulting in a set of SNPs that are replicable in at least three independent test sets. Due to the involvement of literature-identified SNPs and testing in the existing dataset, a certain level of over-fitting is expected in this model. Additional SNPs were selected by an F-test. The evaluation in the leaderboard suggested that the SNPs that were determined by comparing dosage/genotype with ΔDAS28 did not have predictive power on the test data, while SNPs determined by comparing dosage/genotype with ΔDAS28 + baseline may help in both sub-challenges.

Team SBI_Lab

The aim of team SBI_Lab's study was to identify candidate SNPs playing a role in the response to therapy in RA patients by compiling several sources of information. To account for the imbalance between the number of SNPs and the number of patients, they performed a feature selection procedure on the genetic data. First, SNPs were mapped to genes using the Ensembl Variation database[2] and BIOMART service[3]. SNPs that could not be mapped to a gene were discarded. Second, SNPs were prioritized based on association and correlation measures between SNP dosage and patient responses, independently for each drug. The list of candidates was expanded using gene-priorization algorithms that combined protein-protein interaction networks and expression data. The procedure is based on the guilt-by-association principle[4] and we selected from the extended list only those candidates with known SNPs. The resulting SNPs, in combination with clinical data, were used to predict the patients' response to treatments by means of regression-based models[1] and a 10-fold cross-validation on the training dataset. When models were applied to an independent dataset (the leaderboard set), their predictive power decreased significantly, pointing out a problem of overfitting in the model. After comparison of the initial list of potential candidates and the use of external sources of information, we confirmed that the predictive value of the original list of candidate SNPs was not improved by any of the external information. Therefore, the SNP list was reduced by selecting only those showing the highest Pearson's correlation with the patients' response (ΔDAS28) in the leaderboard set. Only a few candidate SNPs out of 70 submitted to the real-time leaderboard for each drug (22 for etanercept, 6 for adalimumab and 15 for infliximab) were used. Although this selection was likely overfitting the training with the leaderboard set, this was irrelevant to testing the approach in a completely independent dataset like CORRONA-CERTAIN. The CORRONA dataset included two drugs that were not present in the training dataset: certolizumab and golimumab. For these two drugs, the infliximab model was used because: 1) both drugs are monoclonal antibodies; and 2) infliximab showed stronger association values compared to adalimumab. The selected SNP dosages were used in combination with clinical data (gender, age, baselineDAS, co-treatment with methotrexate) as features to train different classifiers using WEKA[5] for the training dataset, including linear regression based algorithms, support vector machines, decision trees and neural networks. Amongst all the classifiers tested, Gaussian processes for regression provided the best results in terms of Pearson's correlation between observed and predicted ΔDAS28, both in the training and

the real-time leaderboard test dataset. The prediction of absolute ΔDAS28 (subchallenge 1) was converted into a scored binary prediction (responder/non-responder, subchallenge 2) by refactoring the predicted ΔDAS28 score.

Team Linked Open Data

This team experimented with converting the clinical and genetic datasets into the Resource Description Framework (RDF) format based on existing vocabularies and best practices. Then, they accessed existing Linked Data [http://linkeddata.org/] resources (primarily Bio2RDF[6]) to enrich the patient dataset by linking the graph of patient data to the graph representations of relevant biomedical datasets, including NCBI Gene, PharmGKB, Drug Bank, Gene Ontology and Uniprot. In early submissions, the team used the automated ontology learning tool DL-learner[7] for patient classification, but found that it did not produce satisfying results for this dataset. For later submissions, the team used the Python scikit-learn machine learning framework[8] to classify patients based on clinical parameters and a small set of SNPs derived from findings in existing literature and datasets. For final submissions, they used gradient boosting classifiers with decision trees as base classifiers.

Team Lucia

Team Lucia selected SNPs in two independent steps, based on prior biological knowledge and on statistical criteria. 242 genes were selected from literature that were known to play a role in the development of RA, or to be targets for the drugs used in the considered treatments[9,10]. All SNPs within 10kb upstream and 1 kb downstream of the selected genes, as well as those located in their distant enhancer sequences[11,12], were included. Among these SNPs, those located in introns were discarded. This led to a list of 3,840 SNPs selected on available biological data. SNPs were also ranked by mutual information and the top 3000 were selected for each treatment. This threshold was chosen by cross-validation. This resulted in a single list of 17,896 statistically selected SNPs. The lists of statistically and biologically selected SNPs were combined and used to fit a Support Vector Regression (SVR) model[13] for each different treatment . The kernels used for this SVR consisted in a weighted sum of different kernel for each variable (SNPs, age, sex, disease level). Either the Tanimoto or the MinMax[14] kernel was used for the SNP data, the Dirac kernel was used for the sex variable, and a linear kernel was used for age and baseline disease level.

Team Outliers

A hierarchical regression model was used to account for the dependence of baseline DAS28 on ΔDAS28's variance. First, a gamma distributed generalized linear model (GLM) of baseline DAS28 was used to predict end DAS28 (= baseline DAS28 - ΔDAS28). From then on the scaled residuals of the GLM was used as the transformed outcome instead of the original ΔDAS28 variable. A lasso model of the clinical variables and SNPs was then used to predict the transformed outcome. ΔDAS28 was calculated directly from the GLM's end DAS28 predictions. Non-response probabilities for the classification subchallenge were calculated by assuming that ΔDAS28 for each patient is normally distributed with mean equal to their predicted ΔDAS28 value and variance equal to a rescaled estimate of the lasso model's variance[15]. The genetic model used 160 SNPs associated with TNF, anti-TNF response and methotrexate response, collected from PharmGKB[16] and other participant teams, as features. The optimal model set all SNP coefficients to zero using cross validation, which is equivalent to regularizing out (removing) the contribution of all genetic variants from our final genetic model.

Team STSI

We used a tree-based regression model, known as cubist regression[17], to model deltaDAS as a function of the clinical variables. We then curated sets of features based on genotypes including known SNPs associated with RA presence, SNPs associated with RA response to treatment, eQTLs for TNF-alpha, ancestry estimates, principle components of genotypes and presence of damaging mutations aggregated to biological pathways. However, none of these features improved the performance of our clinical-only model during cross-validation. Therefore, our final model only included clinical variables and no genetic features.

Team TeamMI

TeamMI's solution to the challenge consisted of two parts: feature selection and prediction models. The initial set of genetic features were collected from various sources, namely (i) tests of association with ΔDAS28 and EULAR non-response, after adjusting for clinical covariates such as gender, age, batch, cohort, baseline DAS28, drug and methotrexate co-

therapy; (ii) literature survey[18], (iii) eQTL analysis , and (iv) differential gene expression analysis . The final set of SNPs was manually selected based on the prediction performances on the leaderboard, by including only those SNP sets that resulted in improved performance as compared to the prediction obtained with clinical features alone. Using the selected SNPs and the provided clinical information, two independent prediction models were adopted: (i) a Bayesian sparse linear mixed model implemented in the software package GEMMA[19], and (ii) a Bayesian efficient multiple kernel learning method BEMKL[20]. GEMMA enables modelling of a mixture of a small number of large genetic effects and a large number of small random genetic effects. The BEMKL model encodes the information from different data sources into kernels, and integrates the kernels for the prediction by learning kernel weights and sample weights across different samples. In GEMMA prediction, the SNP data were first projected to make these predictors linearly independent of the clinical variables. In BEMKL prediction, Kronecker delta kernels were used for the categorical features and Gaussian kernels for the other predictors.

Team wtwt5237

According to existing literature, 18 MTX response related SNPs, 124 anti-TNF drug response related SNPs, 78 RA-related SNPs, and 20,385 immune-related SNPs were selected. Principal components (PCs) of RA-related SNPs were calculated by PCA. PCs of all SNPs and immune-related SNPs were calculated by PLINK[21]. For both sub challenges, 5 different algorithms were trained, which include randomForest (alg1), LASSO[22] (alg2), SVM (alg3), Adaboost (alg4) and PLS (alg5). For the quantitative subchallenge, we predicted final DAS28 levels for alg1, alg3 and alg5, and calculated ΔDAS28 by subtracting baseline DAS28 from the predicted final DAS28 levels. In each of the 5 algorithms, we included baseline DAS28, age, gender and MTX usage. Additionally, anti-TNF treatment is used as a covariate for alg1, alg3, and alg4, but for alg2 and alg5, patients were stratified according to the anti-TNF drugs used and made drug-specific training and prediction. Two novel drugs, certolizumab and golimumab, were modeled as adalimumab and infliximab, respectively, according to similarities of these compounds. In addition, team wtwt5237 included different combinations of SNP sets or the first 3-70 PCs of immune-related, RA-related or all SNPs in the machine learning algorithms. Finally, predictions from the 5 algorithms were combined using different weights.

Supplementary References

1. Rasmussen, C. E. & Williams, C. K. I. *Gaussian Processes for Machine Learning*. (The MIT Press, 2006).

2. Flicek, P. *et al.* Ensembl 2014. *Nucleic Acids Res.* **42,** (2014).

3. Guberman, J. M. *et al.* BioMart Central Portal: An open database network for the biological community. *Database* **2011,** (2011).

4. Guney, E. & Oliva, B. Exploiting Protein-Protein Interaction Networks for Genome-Wide Disease-Gene Prioritization. *PLoS One* **7,** (2012).

5. Hall, M. *et al.* The WEKA data mining software: an update. *SIGKDD Explor. Newsl.* **11,** 10–18 (2009).

6. Belleau, F., Nolin, M. A., Tourigny, N., Rigault, P. & Morissette, J. Bio2RDF: Towards a mashup to build bioinformatics knowledge systems. *J. Biomed. Inform.* **41,** 706–716 (2008).

7. Lehmann, J. DL-Learner: Learning Concepts in Description Logics. *J. Mach. Learn. Res.* **10,** 2639–2642 (2009).

8. Pedregosa, F. *et al.* Scikit-learn: Machine Learning in {P}ython. *J. Mach. Learn. Res.* **12,** 2825–2830 (2011).

9. Kanehisa, M. & Goto, S. Kyoto Encyclopedia of Genes and Genomes. *Nucleic Acids Res.* **28,** 27–30 (2000).

10. Law, V. *et al.* DrugBank 4.0: Shedding new light on drug metabolism. *Nucleic Acids Res.* **42,** (2014).

11. Andersson, R. *et al.* An atlas of active enhancers across human cell types and tissues. *Nature* **507,** 455–61 (2014).

12. Chepelev, I., Wei, G., Wangsa, D., Tang, Q. & Zhao, K. Characterization of genome-wide enhancer-promoter interactions reveals co-expression of interacting genes and modes of higher order chromatin organization. *Cell Research* **22,** 490–503 (2012).

13. Smola, A. J. & Schölkopf, B. A tutorial on support vector regression. *Statistics and Computing* **14,** 199–222 (2004).

14. Ralaivola, L., Swamidass, S. J., Saigo, H. & Baldi, P. Graph kernels for chemical informatics. *Neural Networks* **18,** 1093–1110 (2005).

15. Reid, S., Tibshirani, R., Friedman, J. & Jan, M. E. A Study of Error Variance Estimation in Lasso Regression. 1–30

16. Whirl-Carrillo, M. *et al.* Pharmacogenomics knowledge for personalized medicine. *Clin. Pharmacol. Ther.* **92,** 414–7 (2012).

17. Holmes, G., Hall, M., Frank, E. & Zealand, N. in *Advanced Topics in Artificial Intelligence* **1747,** 1–12 (1999).

18. Okada, Y. *et al.* Genetics of rheumatoid arthritis contributes to biology and drug discovery. *Nature* **506,** 376–81 (2014).

19. Zhou, X., Carbonetto, P. & Stephens, M. Polygenic Modeling with Bayesian Sparse Linear Mixed Models. *PLoS Genet.* **9,** (2013).

20. Costello, J. C. *et al.* A community effort to assess and improve drug sensitivity prediction algorithms. *Nat. Biotechnol.* 1–103 (2014). doi:10.1038/nbt.2877

21. Purcell, S. *et al.* PLINK: a tool set for whole-genome association and population-based linkage analyses. *Am. J. Hum. Genet.* **81,** 559–575 (2007).

22. Goeman, J. J. L1 penalized estimation in the Cox proportional hazards model. *Biometrical J.* **52,** 70–84 (2010).

23. Freund, Y. & Schapire, R. E. Large Margin Classification Using the Perceptron Algorithm. *Mach. Learn.* **37,** 277–296 (1999).

24. Landwehr, N., Hall, M. & Frank, E. Logistic Model Trees. *Mach. Learn.* **59,** 161–205 (2005).

25. Sumner, M., Frank, E. & Hall, M. in *Knowledge Discovery in Databases: PKDD 2005* (eds. Jorge, A., Torgo, L., Brazdil, P., Camacho, R. & Gama, J.) **3721,** 675–683 (Springer Berlin Heidelberg, 2005).

26. Platt, J. C. in *Advances in kernel methods* 185–208 (MIT Press, 1999).

27. Zhang, T. Solving large scale linear prediction problems using stochastic gradient descent algorithms. *Proceedings of the twenty-first international conference on Machine learning* 116 (2004). doi:10.1145/1015330.1015332

28. Breiman, L. Random Forests. *Mach. Learn.* **45,** 5–32 (2001).

29. Frank, E. & Witten, I. H. Generating Accurate Rule Sets Without Global Optimization. *Proceedings of the Fifteenth International Conference on Machine Learning* 144–151 (1998).

30. John, G. H. & Langley, P. Estimating continuous distributions in Bayesian classifiers. *Proceedings of the Eleventh conference on Uncertainty in artificial intelligence* 338–345 (1995).

31. Rumelhart, D. E., Hinton, G. E. & Williams, R. J. in *Parallel distributed processing: explorations in the microstructure of cognition, vol. 1* (eds. David, E. R., James, L. M. & Group, C. P. R.) 318–362 (MIT Press, 1986).

32. Friedman, J., Hastie, T. & Tibshirani, R. Additive logistic regression: a statistical view of boosting. 337–407 (2000). doi:10.1214/aos/1016218223

33. Cessie, L. & van Houwelingen, J. C. Ridge Estimators in Logistic Regression. *Appl. Stat.* **41(1),** 191–201 (1992).

34. Cohen, W. Fast Effective Rule Induction. in *In Proceedings of the Twelfth International Conference on Machine Learning* 115–123 (1995). doi:citeulike-article-id:3157878

35. Quinlan, J. R. *C4.5: programs for machine learning*. (Morgan Kaufmann Publishers Inc., 1993).

36. Aha, D. W., Kibler, D. & Albert, M. K. Instance-Based Learning Algorithms. *Mach. Learn.* **6,** 37–66 (1991).
